# A case of invasive lobular carcinoma with signet-ring-cell differentiation treated with multidisciplinary therapy: a rare case report

**DOI:** 10.3389/fonc.2026.1926146

**Published:** 2026-09-09

**Authors:** Haruko Takuwa, Tetsuo Hori, Naoki Goda, Megumi Takeuchi

**Affiliations:** 1 Department of Breast Surgery, Mitsubishi Kyoto Hospital, Kyoto, Japan; 2 Department of Medical Oncology, Mitsubishi Kyoto Hospital, Kyoto, Japan; 3 Department of Diagnostic Pathology, Mitsubishi Kyoto Hospital, Kyoto, Japan

**Keywords:** breast cancer, CDH1, E-cadherin, gastrointestinal metastases, invasive lobular carcinoma with signet-ring-cell differentiation

## Abstract

**Background:**

Invasive lobular carcinoma (ILC) with signet-ring-cell (SRC) differentiation of the breast is a relatively rare disease and is reported to frequently metastasize to the gastrointestinal and urinary systems.

**Case presentation:**

A 66-year-old woman presented for routine breast cancer screening without any subjective symptoms. A mass detected in the upper-outer region of her right breast. Core needle biopsy led to the diagnosis of ILC with SRC of the breast. Positron emission tomography-computed tomography, urine cytology, and upper and lower gastrointestinal endoscopy revealed no abnormal findings, and the disease was diagnosed as cT1cN0M0, Stage I. Immunohistochemical examination showed estrogen receptor (ER)-positive (10%, weak), progesterone receptor (PgR)-negative (0%), human-epidermal growth factor receptor 2 (HER2) score 0, and Ki-67 index of 9.8%, suggesting a luminal B-like subtype. The patient received 4 cycles of epirubicine and cyclophosphamide followed by 4 cycles of docetaxel (DTX) as neoadjuvant chemotherapy (NAC). Because prolonged neutropenia occurred during DTX treatment, temporary treatment interruptions were required; however, all cycles were completed with a relative dose intensity of 80%. As stable disease after completion of NAC, right breast-conserving surgery with sentinel lymph node biopsy was performed. Pathological assessment after neoadjuvant chemotherapy revealed ypT1c (1.8 cm) sN0 (0/1), therapeuthic effect Grade 2b, ER- (0%), PgR- (0%), HER2 0, Ki-67 20% of residual tumor remaining in the breast. Therefore, BRACAnalysis^®^ testing was performed as a companion diagnostic test and was negative. The patient subsequently have received postoperative radiotherapy, and 8 cycles of capecitabine therapy.

**Conclusions:**

In our cohort of ILC patients treated at our institution, we also observed cases with gastrointestinal metastases; therefore, attention should be paid to symptoms and findings suggestive of metastasis to these sites as well.

## Introduction

Breast cancer is one of the most common malignant tumors worldwide (1). Approximately 90% of invasive breast cancers are invasive ductal carcinoma; however, several special histological types are also recognized, including invasive lobular carcinoma (ILC), mucinous carcinoma, apocrine carcinoma, invasive micropapillary carcinoma, tubular carcinoma, medullary carcinoma, metaplastic carcinoma, and adenoid cystic carcinoma.

ILC with signet-ring-cell (SRC) differentiation of the breast is a rare carcinoma, accounting for approximately 0.1-0.3% of all breast cancers, and is considered to originate from rare variant of ILC (2–6). Its prognosis is thought to depend more on the underlying tumor biology than on the presence of ILC with SRC themselves (7). Long-term survival may be achievable in estrogen receptor (ER)-positive/human epidermal growth factor receptor 2 (HER2)-negative cases; however, caution is required because of the tendency for diffuse infiltration, late recurrence, and metastasis to unusual sites such as the peritoneum, gastrointestinal tract, ovaries, urinary tract, and meninges (8–10). In addition, pleomorphic variants and highly proliferative tumors have been reported to be associated with poor prognosis (11, 12). However, the response to breast cancer treatment remains controversial.

Here, we report a case of ILC with SRC of the breast derived.

## Case presentation

**Figure 1:**
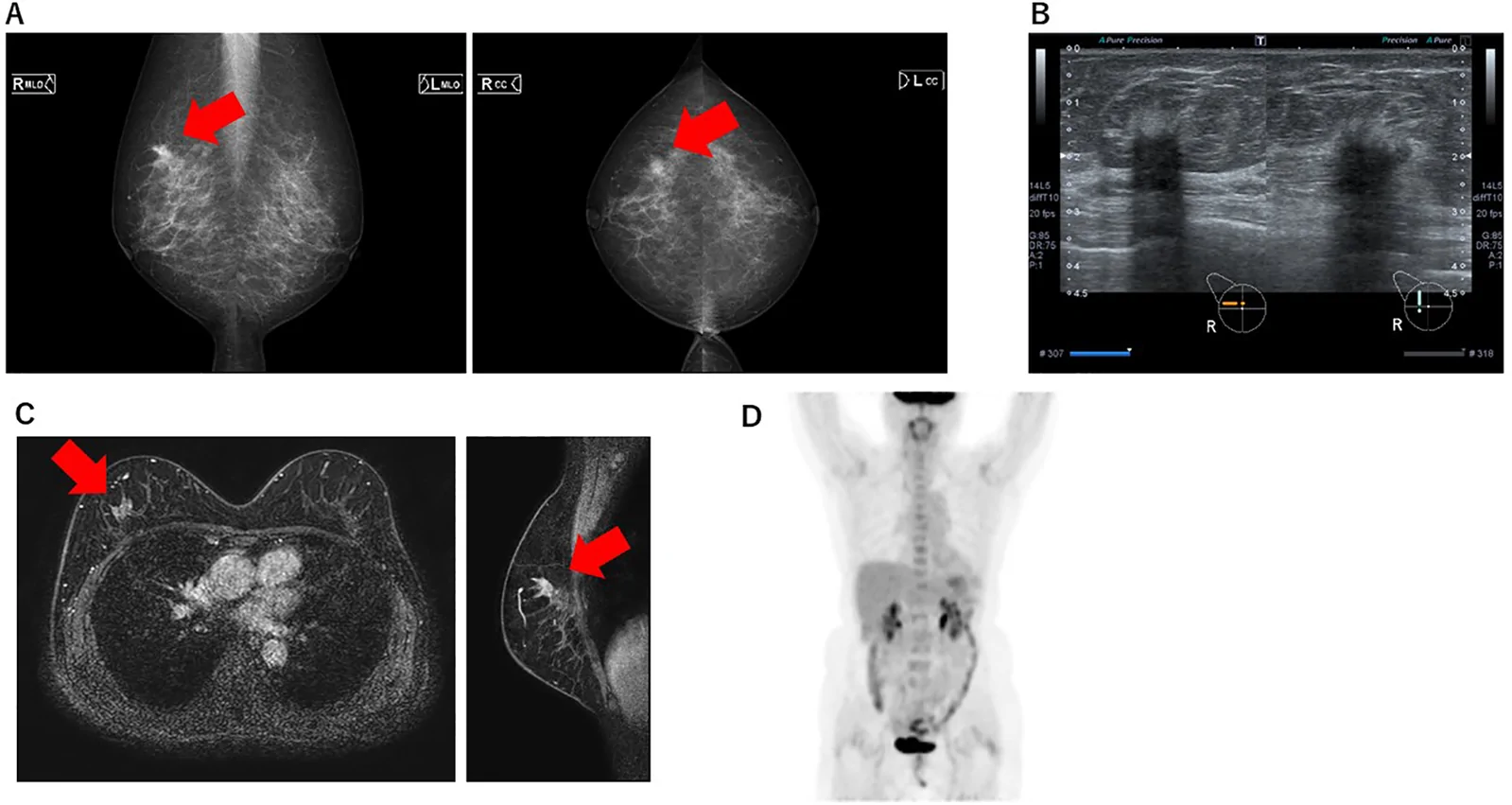
At diagnosis of the right breast cancer. The red arrow indicates the tumor. **(A)** On mammography, an irregular mass with spiculated margins causing architectural distortion was identified in the upper outer quadrant of the right breast. **(B)** Breast ultrasonography demonstrated a hypoechoic mass at the corresponding site with posterior acoustic shadowing and features suggestive of infiltrative growth into the surrounding fatty tissue. **(C)** Magnetic resonance image also revealed a mass with surrounding tissue invasion; however, there was no marked early enhancement on dynamic imaging, and a gradual enhancement pattern was observed. **(D)** On positron emission tomography, no definite distant metastasis were identified. The right breast tumor showed an SUVmax of 1.2 on FDG-PET/CT.

**Figure 2:**
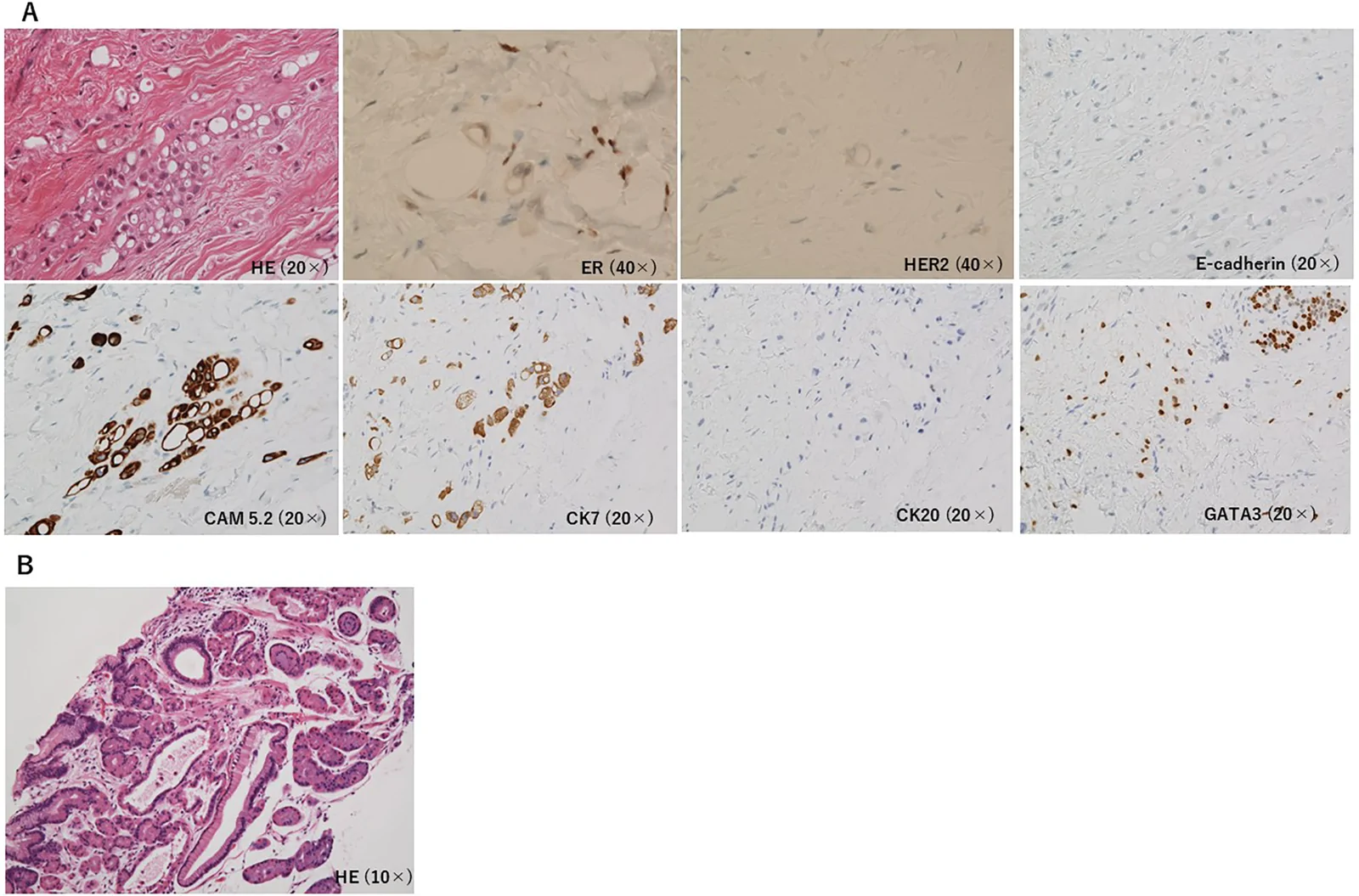
Pathological study at diagnosis. The number in the lower right of each figure indicates the magnification level. **(A)** Histopathological findings from the core needle biopsy at diagnosis are shown. On hematoxylin and eosin staining, atypical cells with eccentrically located nuclei were observed infiltrating in a single-cell pattern, suggesting signet-ring cell carcinoma derived from invasive lobular carcinoma. Signet-ring cell morphology is observed in more than 20% of the tumor cells. Additional immunohistochemical staining showed estrogen receptor (ER) positive (10%, weak), human epidermal growth factor receptor 2 (HER2) negative, E-cadherin negativity, CK7 positivity, and CK20 negativity, GATA3 positivity leading to the diagnosis of primary signet ring cell carcinoma of the breast. HE, hematoxylin-eosin staining; ER, estrogen receptor; HER2, human epidermal growth factor receptor 2. **(B)** During upper and lower gastrointestinal endoscopy, fundic gland polyps were observed. Histopathological examination revealed no malignant findings.

A 66-year-old woman presented for breast cancer screening without any symptoms. The patient had no notable past medical history or family history, including an early-onset gastric cancer. A mass was noted in the right breast, and imaging study revealed irregular shaped mass (**Figures 1A–C**). Core needle biopsy confirmed that the tumor was estrogen receptor (ER) positive (10%, weak), progesterone receptor (PgR) negative (0%), human epidermal growth factor receptor 2 (HER2) negative, Ki-67 9.8% ILC with SRC (**Figure 2A**). Immunohistochemical staining showed CAM5.2 (+), CK7 (+), CK20 (−), E-cadherin (−), GATA3 (+), and a wild-type p53 pattern, leading to the diagnosis of ILC with SRC of breast origin. SRC morphology is observed in more than 20% of the tumor cells. The right breast tumor showed an SUVmax of 1.2 on the 18F-fluorodeoxyglucose positron emission tomography-computed tomography. As previously reported, 18F-fluorodeoxyglucose uptake in SRC components was low (13). The 18F-fluorodeoxyglucose uptake observed in the urinary and gastrointestinal tracts was considered physiological (SUVmax 2.2-2.5) (**Figure 1D**). Urine cytology and upper and lower gastrointestinal endoscopic examinations revealed a fundic glands polyp (**Figure 2B**), and no evidence of distant metastasis was detected. Radiological study classified she was in cT1cN0M0, stage I disease (UICC-TNM classification 9th edition).

**Figure 3:**
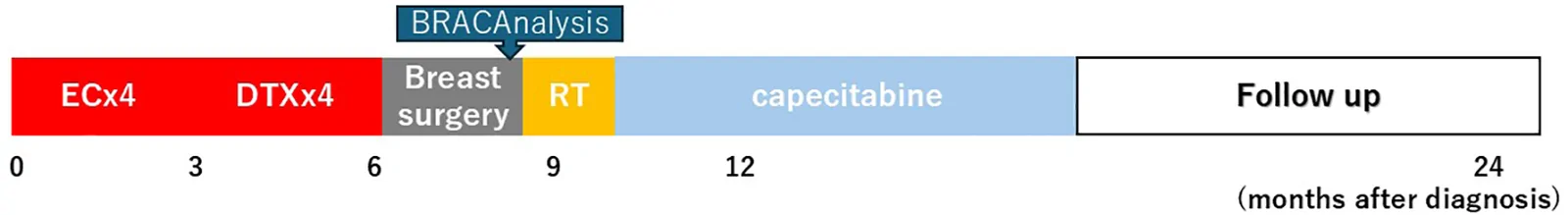
Clinical time line of the patient. The patient underwent approximately 6 months of neoadjuvant chemotherapy followed by breast-conserving surgery. After completing postoperative radiotherapy, she received 6 months of adjuvant capecitabine.

Although the present case showed a low Ki-67 labeling index, it was considered to have low endocrine responsiveness and, given its T1c stage, to be an appropriate candidate for adjuvant chemotherapy. Neoadjuvant chemotherapy (NAC) was selected because, if a pathological complete response was not achieved, additional postoperative treatment options to reduce the risk of recurrence would be available. Four cycles of epirubicin and cyclophosphamide followed by four cycles of docetaxel (DTX) were administered as NAC (**Figure 3**). Although prophylactic pegfilgrastim was administered, treatment from the second cycle of DTX onward was completed at 4-week intervals because of neutropenia, resulting in a relative dose intensity of 80%. Residual tumor was still detected in the right breast after completion of NAC, and the imaging study showed response to treatment was assessed as stable disease (**Supplementary Figure 1**).

**Figure 4:**
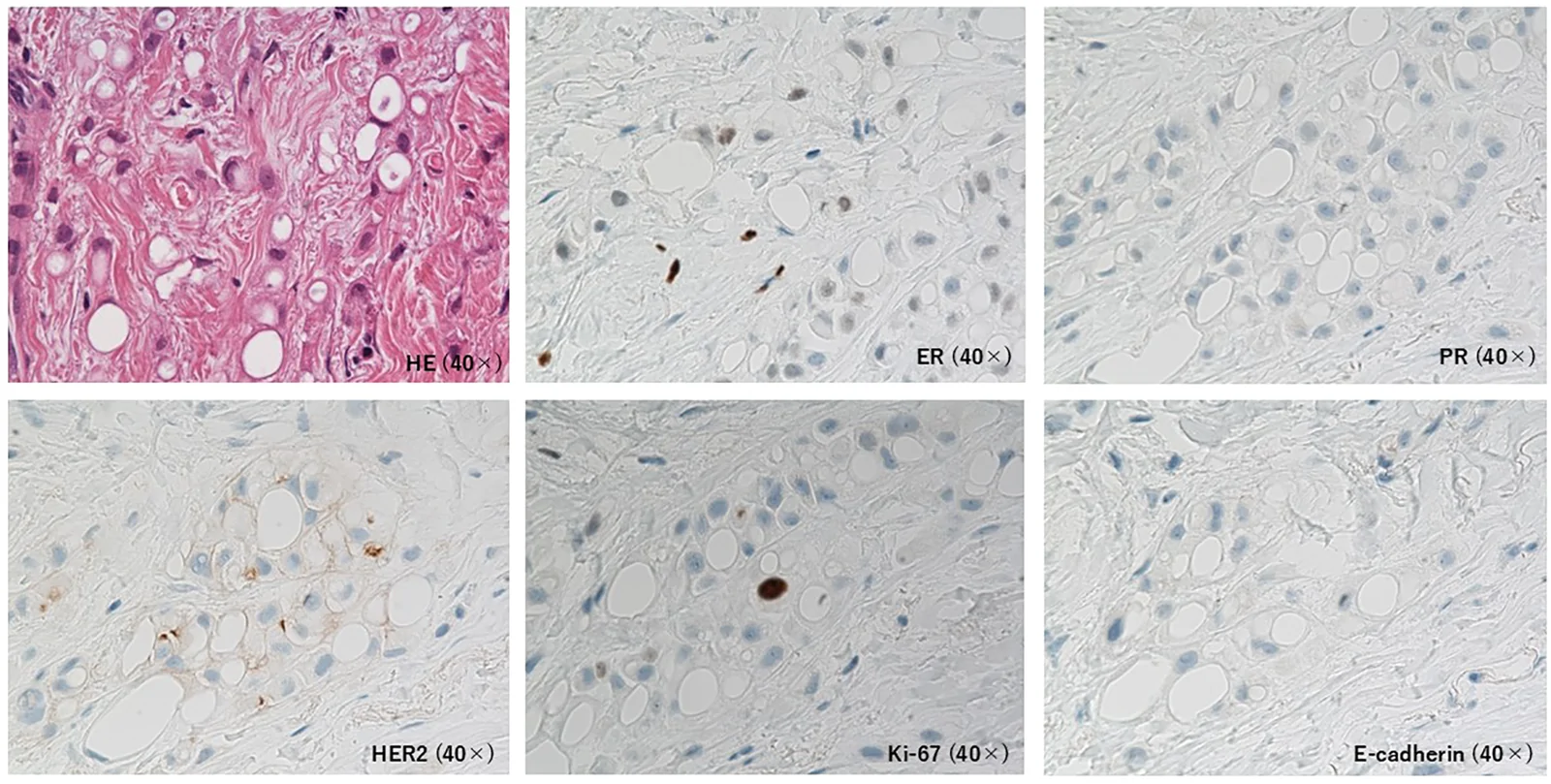
Pathological study after surgery. The number in the lower right of each figure indicates the magnification level. Postoperative histopathological analysis demonstrated that the residual tumor, however viable tumor cells were scarcely detectable. Among the remaining tumor cells, fewer than 20% are estimated to show signet-ring cell morphology. The tumor, originally ER-positive (10%, weak expression), had converted phenotypically to triple-negative breast cancer; estrogen receptor (ER) turned to negative (0%), progesterone receptor (PgR) negative (0%), human epidermal growth factor receptor 2 (HER2) negative, Ki-67 20%. The therapeutic response was evaluated as Grade 2b. HE, hematoxylin-eosin staining; ER, estrogen receptor; PgR, progesterone receptor; HER2, human epidermal growth factor receptor 2.

The patient underwent breast-conserving surgery of the right breast and sentinel lymph node biopsy. Pathological examination revealed ypT1c (1.8cm) N0 (0/1), therapeutic effect Grade 2b, ER-negative (0%), PgR-negative (0%), HER2 score 0, and Ki-67 index of 20% (**Figure 4**). No lymphatic invasion or vascular invasion was identified, and the surgical margins were negative. The residual cancer burden (RCB) assessment after neoadjuvant chemotherapy was RCB-II, 1.683 (14). Although the tumor was ER-positive at diagnosis (10%, weak staining), it became ER-negative (0%) after NAC. Adjuvant radiotherapy was delivered to the conserved breast (42.56Gy in 16 fractions). Given the presence of residual triple negative breast cancer (TNBC), BRACAnalysis testing was performed to assess eligibility for poly (adenosine diphosphate ribose) polymerase inhibitors, and the results were negative for pathogenic *BRCA1/2* variants. Because a pathological complete response was not achieved following neoadjuvant chemotherapy, the patient opted to receive eight cycles of adjuvant capecitabine for recurrence risk reduction. In ER-positive breast cancer, endocrine therapy is generally expected to provide clinical benefit when ≥1% of tumor cells are ER-positive, even if ER expression is weak. After discussion of the potential benefits and disadvantages, including adverse effects, the patient elected not to receive endocrine therapy.

## Methods

A retrospective cohort study was conducted on 56 patients with ILC, including the present case, who were treated at our institution between 2005 and 2025. Clinical course, treatment outcomes, and prognosis were retrospectively evaluated. A pathological review of the diagnostic core needle biopsy specimens, surgical specimens, and recurrent tumor specimens was performed by a pathologist, with re-evaluation of cases containing tumor cells exhibiting SRC morphology. To evaluate the efficacy of initial treatment, patients with unresectable locally advanced disease were excluded. The median follow-up period was 84 months (range, 12.7–257.3 months).

The ethics committee of our institution approved this study and the use of the details in the patients’ medical records (25-34).

## Discussions

SRC in the mammary gland is considered to be more strongly associated with ILC in particular (2–7). From a diagnostic standpoint, it is extremely important to distinguish these lesions from metastases originating from other organs, such as gastric carcinoma. However, most cases are of the luminal subtype, showing ER-positivity and HER2-negativity, and treatment is generally recommended to follow standard breast cancer management algorithms (15). On the other hand, Cai et al. used the Surveillance, Epidemiology, and End Results Program database to predict survival outcomes in patients with ILC with SRC and reported that postoperative radiotherapy does not provide additional benefit to these patients (16).

In this case, the primary lesion was a breast mass. The tumor was ER positive, and no definite primary lesion was identified on systemic evaluation. Based on findings including GATA3 positivity and CK7 positivity (3, 17), it was diagnosed as ILC with SRC of the breast, and treatment was initiated accordingly. Compared with typical TNBC and luminal B-like breast cancer with TNBC-like characteristics (18–21), the tumor was expected to exhibit a lower Ki-67 index and decreased chemosensitivity. Nevertheless, its low hormone sensitivity did not justify the omission of chemotherapy from perioperative treatment. Moreover, evaluating the efficacy of NAC would provide valuable information for determining the optimal postoperative adjuvant therapy. Accordingly, neoadjuvant chemotherapy was initiated. Due to prolonged myelosuppression, the patient completed preoperative chemotherapy with DTX at 80% of the planned dose intensity. Because residual tumor was identified in the surgical specimen, treatment options to reduce the risk of recurrence were considered. As the Ki-67 labeling index at diagnosis was relatively low, the efficacy of oral cytotoxic chemotherapy was expected. Adjuvant capecitabine was administered postoperatively (22–25). Owing to myelosuppression, capecitabine was administered at a reduced dose below the recommended level, and the 8 cycles of treatment was completed without any notable severe adverse events. At the current follow-up, 2 years after surgery, no signs of recurrence have been observed. Careful long-term surveillance will be continued, as the current follow-up is too short to make any conclusion about late recurrence.

Kioleoglou et al. reviewed cases of ILC with gastrointestinal metastases and reported that, among the four patients who had stage I disease at the time of primary diagnosis, two had not received adjuvant systemic therapy including one patient who was definitively diagnosed with TNBC (7). The metastatic patients may have been associated with insufficient adjuvant systemic treatment. Although ILC with SRC has frequently been reported to exhibit treatment resistance and an unfavorable prognosis following recurrence or metastasis, curative treatment may still be achievable when the disease is diagnosed at a stage characterized by a relatively low tumor burden or a low risk of recurrence (13). Accordingly, active therapeutic intervention is warranted in such patients.

**Table 1** summarizes the clinical characteristics of the 56 patients with operable ILC who underwent treatment at our institution. Histopathological review revealed that 11 patients with ILC, including the present case, harbored tumors containing 5–60% tumor cells with SRC morphology. **Table 2** presents the clinical characteristics of patients with metastatic disease.

**Table 1:** Patient characteristics of the 56 patients with operable ILC who underwent treatment at our institution.

	N = 56
Age at diagnosis(y.o)(range)	62 (41-89)
Stage at diagnosis, No. (%)	
I	22 (39.3)
IIA	11 (19.6)
IIB	11 (19.6)
IIIA	8 (14.3)
IIIB	3 (5.4)
IIIC	1 (1.8)
Subtype, No. (%)	
ER+/HER2-	47 (83.9)
ER+/HER2+	0 (0.0)
ER-/HER2+	2 (3.6)
ER-/HER2-	6 (10.7)
Unknown	1 (1.8)
Histrogical grade	
1	6 (10.7)
2	42 (75.0)
3	3 (5.4)
Unknown	5 (8.9)
Containing signet-ring-cell component (5-60%) (%)	20 (35.7)
Ki-67 (%, range)	15.8 (2.0-72.0)
*BRCA1/2* pathogenic variant (%)	1 (1.8)
Synchronous/metachronous bilateral breast cancer	4 (7.2)
Breast surgery	
Breast conserving surgery	21 (37.5)
Mastectomy	35 (62.5)
Axillary surgery	
Sentinel lymph node biopsy	38 (67.9)
Axillary lymph node dissection	18 (32.1)
Chemotherapy	20 (35.7)
Anthracycline	15 (75.0)
Taxane	19 (95.0)
With immune checkpoint inhibitor (pembrolizumab)	1 (5.0)
Radiation therapy	30 (53.6)
Endocrine therapy	48 (85.7)
monotherapy	34 (70.8)
With oral 5-FU (capecitabine, TS-1, UFT)	10 (20.8)
With CDK4/6i (abemaciclib)	4 (8.3)
Distant metastasis, No. (%)	9 (16.1)

Consistent with previous reports, the majority of tumors were ER-positive/HER2-negative, and many exhibited a relatively low Ki-67 proliferation index. Nine patients (16.1%) developed postoperative metastases.

ILC, invasive lobular carcinoma; ER, estrogen receptor; HER2, human epidermal growth factor receptor 2; 5-FU, 5-fluorouracil; TS-1, tegafur, gimeracil and oteracil; UFT, tearful and uracil; CDK4/6i, cyclin-dependent kinase 4 and 6 inhibitors.

**Table 2:** Clinical characteristics of the patients with metastatic disease.

	Age at diagnosis	Stage	Signet-ring-cell component(%)	Subtype of primary tumor	CT/RT/ET	Site of 1^st^ metastatic legion	DFS (months)
1	51	cT1cN1M0	0	ER+/HER2-	-/+/+	bone	5.6
2	80	cT1cN1M0	0	ER-/HER2-	-/-/-	bone	16.8
3	42	cT2N0M0	0	ER+/HER2-	+/-/+	Local recurrence	80.5
4	76	cT2N0M0	60	ER+/HER2-	-/-/+	bone	25.6
5	44	cT2N1M0	0	ER+/HER2-	-/-/+	Ovary, peritoneal	199.2
6	51	cT2N2M0	0	ER+/HER2-	+/-/+	contralateral breast	7.7
7	75	cT2N2M0	30	ER-/HER2+	-/+/-	bone	4.1
8	61	cT3N0M0	0	ER+/HER2-	-/-/+	Gastric mucosa, local	43.6
9	45	cT3N2M0	0	ER+/HER-	+/-/+	Gastric mucosa	194.1

Case 8 and 9 developed gastrointestinal recurrence, which occurred as a late recurrence 3 and 16 years, respectively.

ILC, invasive lobular carcinoma; CT, chemotherapy; RT, radiation therapy, ET, endocrine therapy; DFS, disease free survival; ER, estrogen receptor; HER2, human epidermal growth factor receptor 2.

Among the patients with ILC with SRC in the primary tumor, Case 4 and 7 developed metastases. Histopathological examination confirmed that 60% and 30% respectively of the primary tumor consisted of SRC components in these patients. Case 4 developed multiple bone metastases two years after surgery while receiving adjuvant endocrine therapy. Because case 7 had locally advanced HER2-enriched breast cancer, intensive perioperative systemic therapy would ordinarily have been recommended. However, this treatment could not be administered because of her underlying medical condition, and recurrence occurred shortly after completion of radiotherapy. Among the other patients with SRC, although there was considerable heterogeneity in tumor and patient characteristics, none developed recurrence. However, because the follow-up period was relatively short, ranging from 2 to 10 years, continued follow-up is warranted.

Two patients developed gastric mucosal metastasis (**Table 2**, case 8, 9). Although the incidence of gastrointestinal metastases from ILC is relatively low at 3.5% (2/56), considering that gastrointestinal metastases from conventional invasive ductal carcinoma occur even less frequently, these findings suggest that ILC carries a higher risk of gastrointestinal metastasis among breast cancer subtypes. Case 8 was unable to receive adequate perioperative chemotherapy or radiotherapy because of financial constraints and developed locoregional recurrence and gastric submucosal metastasis less than 4 years after surgery. No SRC components were identified in either the primary tumor or the metastatic lesion. Because case 9 had locally advanced ER-positive/HER2-negative breast cancer, current treatment would include adjuvant endocrine therapy with a 2-year course of a cycle-dependent kinase 4 and 6 (CDK4/6) inhibitor in addition to intensive chemotherapy. However, this treatment option was not available at the time of diagnosis, and therefore no CDK4/6 inhibitor was administered. The patient developed recurrence 194 months after surgery, following completion of 10 years of adjuvant endocrine therapy. The patient experienced late recurrence and demonstrated favorable endocrine sensitivity, as evidenced by a good response to first-line treatment with anastrozole plus abemaciclib. These findings suggest that gastrointestinal luminal metastasis, although uncommon, may occur in ILC in general. Previous studies have reported that gastric metastases from breast carcinoma more frequently demonstrate the uninvacuolated type of SRC than primary gastric carcinoma. However, this feature was not observed in the present case, including in the primary breast lesion (26).

Although clinicopathological evidence remains limited, gastrointestinal and urinary luminal metastasis has been reported in subset of patients with ILC (7–9, 27–30). Even when gastrointestinal metastasis develops, disease control after recurrence may be relatively favorable in patients with late recurrence and preserved endocrine sensitivity. In contrast, patients with early recurrence or endocrine-resistant disease are likely to have a poorer prognosis. Therefore, the detection and management of such recurrences represent an urgent clinical issue. This is because, when metastatic recurrences in the gastrointestinal tract or urinary tract are diagnosed only after the onset of symptoms, they can cause significant impairment of digestive and excretory functions, leading to deterioration of the patient’s general condition and potentially making treatment more difficult. Long-term follow-up may be important because recurrence can occur after a prolonged disease-free interval.

In addition, even when SRC carcinoma originates from the stomach, there are many reports indicating that a favorable response can be achieved through appropriate treatment selection based on biomarker expression status (31–33). This suggests the importance of making a diagnosis at an earlier stage, when tumor burden is lower, and selecting the most appropriate therapeutic strategy.

Previous reports have shown that both ILC with SRC and ILC with gastrointestinal metastases exhibit a wide range of phenotypes. In the present case, we examined for pathogenic variants in *BRCA1/2* as a companion diagnostic for poly (adenosine diphosphate ribose) polymerase inhibitors. However, the prevalence of pathogenic *BRCA1/2* variants among patients with ILC at our institution is not particularly high (**Table 1**). On the other hand, given the loss of E-cadherin, it may also be important to assess for pathogenic variants in *CDH1*, the causative gene of hereditary diffuse gastric cancer (HDGC) (34). None of our patients met the diagnostic criteria for HDGC established by the International Gastric Cancer Linkage Consortium (35). Nevertheless, it has been reported that 39–52% of women with hereditary diffuse gastric cancer develop lobular breast cancer (36).

Although the optimal postoperative surveillance strategy for rare special subtypes of breast cancer has not yet been fully established, continued accumulation and application of clinical evidence are expected to facilitate the provision of appropriate surveillance, diagnostic evaluation, and treatment for all patients with breast cancer.

## Conclusions

Compared with conventional breast cancer, ILC with SRC has a greater tendency to metastasize to unusual sites and is frequently reported to show poor responsiveness to treatment after recurrence.

In the present case, systemic therapy was administered based on the tumor phenotype, which had become similar to that of TNBC. In our cohort of ILC patients treated at our institution, we also observed cases with gastrointestinal metastases; therefore, attention should be paid to symptoms and findings suggestive of metastasis to these sites as well.

## Data Availability

The original contributions presented in the study are included in the article/**Supplementary Material**, further inquiries can be directed to the corresponding author/s.

## Supplementary material

Supplementary Figure 1 Imaging study after completion of neoadjuvant chemotherapy. The red arrow indicates the tumor. **(A)** Breast ultrasonography demonstrated a residual hypoechoic mass at the corresponding site with posterior acoustic shadowing and features suggestive of infiltrative growth into the surrounding fatty tissue. **(B)** Magnetic resonance image also revealed a mass with surrounding tissue invasion; however, there was no marked early enhancement on dynamic imaging, and a gradual enhancement pattern was observed.
